# Outcomes of Endolymphatic Sac Surgery for Meniere's Disease with and without Comorbid Migraine

**DOI:** 10.1155/2021/7987851

**Published:** 2021-12-24

**Authors:** Norman A. Orabi, Brian M. Kellermeyer, Christopher A. Roberts, Stephen J. Wetmore, Adam M. Cassis

**Affiliations:** ^1^West Virginia University School of Medicine Department of Otolaryngology, P.O. Box 9200, Morgantown, WV 26506, USA; ^2^Arizona Hearing & Balance Center, 225 S Dobson Rd#1, Chandler, AZ 85224, USA

## Abstract

**Purpose:**

To explore outcomes of endolymphatic sac surgery for patients with Meniere's disease with and without the comorbid condition of migraine.

**Materials and Methods:**

A retrospective chart review of adult patients undergoing endolymphatic sac surgery at a single tertiary care center from 1987 to 2019 was performed. All adult patients who failed medical therapy and underwent primary endolymphatic sac surgery were included. The main outcome measures were vertigo control and functional level scale (FLS) score. Patient characteristics, comorbidities, and audiometric outcomes were tracked as well.

**Results:**

Patients with Meniere's disease and migraine had a stronger association with psychiatric comorbidities (64.29% vs. 25.80%, *p*=0.01), shorter duration of vertigo episodes (143 vs. 393 min, *p*=0.02), and younger age (36.6 vs. 50.8 yr, *p*=0.005) at the time of endolymphatic sac surgery. Postoperative pure tone averages and word recognition scores were nearly identical to preoperative baselines. Class A vertigo control (47.92%) was most common, followed by class B vertigo control (31.25%). The FLS score improved from 4.2 to 2.8 (*p* < 0.001). Both patients with and without migraine had classes A-B vertigo control (66.67% vs. 80.95%) without any statistically significant difference (*p*=0.59). Of the patients who required secondary treatment (10.42%), none had migraine.

**Conclusions:**

Endolymphatic sac surgery is an effective surgical intervention for Meniere's disease with and without migraine. Patients with comorbid migraine tend to be younger and present with psychiatric comorbidities.

## 1. Introduction 

Meniere's disease is a rare condition with a prevalence of 190 per 100,000 but has detrimental effects on function and quality of life [1]. The condition is defined by (1) two or more spontaneous episodes of vertigo lasting 20 minutes to 12 hours, (2) at least one occasion of audiometrically documented low-to-medium frequency sensorineural hearing loss in the affected ear before, during, or after one of the episodes of vertigo, (3) fluctuating aural symptoms (hearing, tinnitus, or fullness) in the affected ear, and (4) another vestibular diagnosis not better accounted for [2]. Although the etiology is unclear, it has been associated with anatomical changes in the inner ear from increased endolymph, which is termed endolymphatic hydrops. The association with endolymphatic hydrops supports the theory that the episodic symptoms are due to endolymphatic duct distention leading to microtears in Reisner's membrane and influx of toxic levels of potassium-rich endolymph [2]. Individual patients often present with a unique constellation of symptoms. For example, two studies by Frejo et al. have used cluster analysis to identify 5 clinical subgroups of Meniere's disease, which includes the Type 4 subgroup that is characterized by comorbid migraine [3, 4]. Moreover, these symptoms overlap with other clinical entities making the diagnosis elusive. The American Academy of Otolaryngology (AAO) published several guidelines to assist in the diagnosis, evaluation, and treatment of Meniere's disease [2, 5].

Initial treatment consists of medical management with low salt diet, diuretics, and betahistine. Antiemetics, vestibular suppressants, and corticosteroids are reserved for symptomatic control during acute episodes. Unfortunately, more aggressive treatment may be necessary for those who continue to have disabling vertigo. Additional treatment consists of intratympanic injection of corticosteroids or gentamicin or surgical options including endolymphatic sac surgery (ESS), vestibular nerve section, and labyrinthectomy. Each treatment option carries its own risks and morbidity. For example, labyrinthectomy is the gold standard for vertigo control but is a destructive procedure resulting in a total loss of hearing [6]. ESS is effective with AAO classes A-B vertigo control of 64.5–77% and minimal risk to hearing [7–9].

Migraine affects about 15.3% of the general population and is characterized by head pain, nausea, vomiting, and sensitivity to environmental stimuli [10]. It is a common comorbidity with Meniere's disease, affecting about 43–56% of these patients [11, 12]. A subset of classical migraine patients develop vestibular migraine, which is often underdiagnosed due to overlapping symptoms with other vestibular disorders such as Meniere's disease [13, 14]. Vestibular migraine has only relatively recently been recognized as a cause of episodic vertigo and did not have a consensus definition by the Barany Society until 2012 [15]. Moreover, vestibular migraine can be accompanied by otologic symptoms (tinnitus, aural fullness, and hearing loss), and it is not uncommon for patients to have both Meniere's disease and vestibular migraine [2, 16]. Some authors have proposed that migraine and Meniere's disease may even be variants of one other and share a common pathophysiological origin through disorders of ionic regulation [17, 18]. When there is uncertainty in the diagnosis or concern for concurrent vestibular migraine, noninvasive medical therapy should be pursued prior to surgical intervention and can be quite effective [2, 14]. Despite the symptoms of migraine and potential to develop vestibular migraine, no studies exploring the effect of migraine on outcomes of ESS in patients with Meniere's disease have been reported. Given the variable outcomes of ESS, we aim to explore the outcomes of ESS in those with a preexisting diagnosis of migraine. We hypothesize that patients with preexisting migraine diagnosis may have a worse outcome with ESS compared to those without migraine.

## 2. Methods

Our study was approved by our university's Institutional Review Board. We conducted a retrospective chart review of all adult patients (≥18 years) with Meniere's disease who underwent ESS at a single tertiary care center from 1987 through 2019 after failing medical therapy. All included patients retrospectively fulfilled Barany Society diagnostic criteria for Meniere's disease. Patients were excluded if they did not undergo ESS or underwent ESS at an outside institution. All surgeries were ESS with mastoid shunt using an arrow-shaped piece of silastic inserted into the sac with the tail of the arrow extending into the mastoid cavity performed by three fellowship trained neurotologists. Seventy-six patients were identified and underwent chart review including paper and electronic medical records. A power analysis was deferred, given that we were able to review the entire available cohort of 76 patients.

Preoperative demographics, comorbidities, and baseline characteristics were collected for all patients. Due to the long time period of data collection extending well before the diagnosis of vestibular migraine, any form or variant of migraine was recorded as positive if present in the past medical history with diagnosis made by either surgeon, primary care provider, or neurologist. Migraine treatment history was not obtained due to the large number of included patients coming from a time period before the knowledge of vestibular migraine and was not routinely available in the records. Baseline functional level scale (FLS) score and Meniere's stage were assessed as per the 1995 AAO Guidelines for Meniere's disease. The FLS score was determined via self-assessment on a standard form or by patient history during clinic visits. Meniere's staging was based on the four-tone average of the pure tone thresholds at 0.5, 1, 2, and 3 kHz of the worst audiogram during the 6-month period before ESS.

Paired preoperative and postoperative pure tone averages (PTAs) and word recognition scores (WRSs) were compared to assess hearing outcomes. Most postoperative audiograms were from the 18–24-month period. If unavailable, we included the postoperative audiogram within 12–36 months that was closest to the preferred 18–24-month postoperative period. There were 42 paired preoperative and postoperative audiograms available for analysis.

Vertigo control grade was used to assess the efficacy of ESS. As per the 1995 AAO Guidelines for Meniere's disease, vertigo control grade classes are divided into classes A–F by dividing the number of definitive vertigo spells in the 6-month period prior to treatment by the number of definitive vertigo spells in the 18–24-month posttreatment period. Classes are defined as *A* = 0%, *B* = 1–40%, *C* = 41–80%, *D* = 81–120%, and *F* > 120%. Of the 76 patients, 28 patients were excluded due to inadequate data in the 18–24-month posttreatment period. Good and poor vertigo controls were defined as vertigo classes A-B and vertigo classes C–F, respectively.

Of note, our patient population had an overlap with a previous descriptive study on primary ESS for the treatment of MD [7]. Unlike the previous study, we focused on preoperative characteristics and postoperative outcomes for primary ESS with migraine as an independent variable. Moreover, our patient population was larger due to the addition of more recent patients since the prior study was completed.

Statistical analysis was performed using RStudio Version 1.4.1103 (© 2009–2021 RStudio, PBC). Descriptive statistics were used for patient demographics. Univariate analysis with a *p* value set at *p*=0.05 was used to analyze the remainder of the data with a subset analysis based on the comorbidity of migraine as an independent variable. We used the two-sample *t*-test for continuous variables and Fisher's exact test for categorical variables.

## 3. Results

Demographics and baseline characteristics were analyzed for 76 patients based on the presence or absence of migraine as shown in Table 1. Meniere's disease with migraine was designated MD + M and Meniere's disease without migraine as MD − M. The average age at the time of surgery was lower at 36.6 years in MD + M compared to 50.8 years in the MD − M group (*P*=0.005). There was a slight female predominance among all patients due to increased prevalence in MD + M patients. Psychiatric comorbidities were present in 64.29% of MD + M patients compared to 25.80% of MD − M patients (*P*=0.01). Comorbidities were anxiety, depression, and panic attacks. The majority of patients in both groups were treated by diuretics, followed by diuretics with either oral or intratympanic steroids. In the MD − M group, there were four patients who received other forms of treatment: two patients received diuretics, intratympanic steroids, and betahistine; one patient received diuretics and used the Meniett device; and one patient received diuretics, oral and intratympanic steroids, and intratympanic gentamycin. Audiometric baselines were similar in MD + M and MD − M patients. Meniere's stage 3 comprised the majority of patients in both groups. The average FLS score was 4.0 and 4.3 in MD + M and MD − M patients, respectively (*p*=0.5). The time from diagnosis to surgery was 47.43 and 52.00 months in the MD + M and MD − M patients, respectively (*p*=0.74).

Analysis of the 42 paired audiograms demonstrated stable hearing outcomes without any significant difference (Table 2). Regarding vertigo control, there were 48 patients with appropriate follow-up in the postoperative 18–24-month period. Overall, ESS decreased the frequency of major vertigo episodes (16.0 vs. 2.4 episodes per month, *p* < 0.001) and FLS score (4.2 vs. 2.8, *p* < 0.001). Class A vertigo control (47.92%) was most common, followed by class B vertigo control (31.25%) (Table 2). Five patients required secondary treatment: two patients underwent revision ESS followed by labyrinthectomy, one vestibular nerve section, one revision ESS followed by vestibular nerve section, and one revision ESS. Decision for revision surgery was based on a relapse of vertigo symptoms after temporary improvement following the initial ESS.

**Figure 1:**
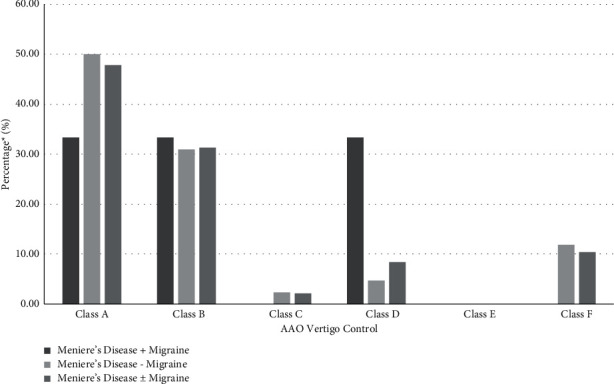
Vertigo outcomes following endolymphatic sac surgery. ^*∗*^Based on specific population sizes for each respective patient category. See Tables 2 and 3 for numerical representation of results.

**Figure 2:**
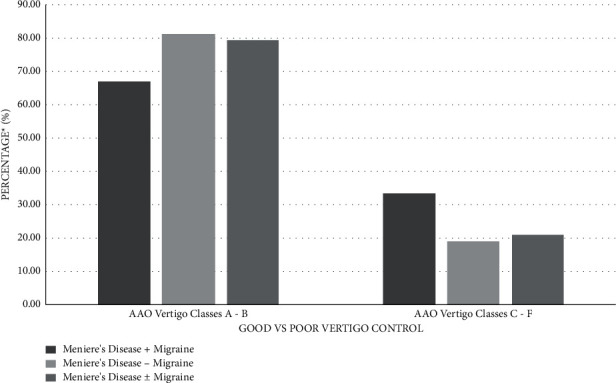
Vertigo control following endolymphatic sac surgery. Good control was defined as AAO classes A-B. Poor control was defined as AAO classes C–F. ^*∗*^Based on specific population sizes for each respective patient category. See Tables 2 and 3 for numerical representation of results.

Six of the 48 patients had MD + M as shown in Table 3. MD + M patients had a shorter duration of major vertigo episodes than MD − M patients (143 vs. 393.4 min, *P*=0.02). Mean vertigo control percentage was similar in MD + M and MD − M patients (*p*=0.96) (Figure 1). The MD + M group had 33.33% of MD + M with class A control and 33.33% with classes C–F control compared to 50.00% with class A control and 19.05% with classes C–F control in the MD − M group. Both groups had similar postoperative FLS scores and classes A-B vertigo control without any statistically significant differences (*p*=0.09 and *p*=0.59) (Table 3 and Figure 2). None of the patients who underwent a secondary procedure were in the MD + M group.

## 4. Discussion

Meniere's disease has detrimental effects on function and quality of life. Although most cases can be managed with medical therapy, a significant proportion of cases require surgical treatment. Migraine is a common comorbidity in these patients. Given the variable outcomes of ESS and possibility of coexisting vestibular migraine, presence of migraine could factor in when considering ESS. Our study is the first to assess the effect of migraine on preoperative characteristics and postoperative outcomes for ESS. We discovered several differences in preoperative characteristics and confirmed the relatively high efficacy of ESS in both MD + M and MD − M patients.

There were several key differences between MD + M patients and MD − M patients. In our study, MD + M patients were more than twice as likely to have psychiatric comorbidities than MD − M patients (Table 1). It is well known that there is a higher association of psychiatric disorders with both Meniere's disease and migraine compared to rates in the general population [19, 20]. Interestingly, vestibular migraine has higher rates of psychiatric comorbidity than Meniere's disease [21]. One explanation for the increased prevalence of psychiatric disorders would be that a subset of our MD + M patients was affected by vestibular migraine given their history of classical migraines, which often predate the vertiginous symptoms of vestibular migraine.

MD + M patients were about 14.2 years younger on average at the time of surgery. Meniere's disease has its highest prevalence in the 5^th^ and 6^th^ decades compared to 4^th^ and 5^th^ decades for migraine, which is consistent with our finding of younger average age for those patients with Meniere's disease and migraine [2, 10, 22]. Although both migraine and Meniere's disease have a female predominance, migraine has a greater association with females as seen in large-scale epidemiological studies [1, 19]. Prior studies on ESS do not consistently demonstrate a female predominance in their surgical candidates [7, 9, 23, 24]. There was a higher female prevalence in MD + M patients but not in MD − M patients. This finding was not statistically significant, but our data are limited by our small population sizes. Vestibular migraine patients are younger and more likely to be female compared with patients with Meniere's disease [2]. These observations in the MD + M group fit the typical demographics for what we now know is vestibular migraine which was not known at that time. There is a possibility that this migraine subset of patients had elements of vestibular migraine contributing to their dizziness.

For patients with Meniere's disease refractory to medical management, more invasive treatment strategies can be pursued including intratympanic injection of corticosteroids or gentamicin, ESS, vestibular nerve section, and labyrinthectomy. ESS is an attractive option because it avoids the total hearing loss and permanent vestibular dysfunction seen with labyrinthectomy. On the other hand, intratympanic gentamycin allows the patient to avoid the morbidity of a surgical procedure but has the potential to damage hearing and vestibular function [25]. Previous studies on ESS have demonstrated excellent vertigo control and improvements in FLS scores. Following ESS, classes A-B vertigo control ranged from 64.5 to 77% and FLS decreased by an average of 0.8–2.0 points [7, 9, 26, 27]. Moreover, a study by Gibson et al. demonstrated similar classes A-B vertigo control between patients receiving ESS and those receiving intratympanic gentamycin injections (73.1% vs. 66.8%, *p*=0.76). However, chronic posttreatment unsteadiness was encountered more frequently in the patients receiving intratympanic gentamycin injections compared to patients receiving ESS (25.0% vs. 0%, *p*=0.009) [25]. Overall, we had classes A-B vertigo control in 79.17% of patients and decreased FLS from 4.2 to 2.8. Our results demonstrated similar efficacy to other studies in the literature.

Migraine is a common comorbidity with Meniere's disease and may confound the decision to pursue ESS. Yet, there are no studies of exploring the effect of comorbid migraine on ESS. Our study demonstrated that 66.67% of MD + M patients had classes A-B control, which is similar to MD − M patients (Table 3, Figure 2) and the aforementioned studies on ESS. Moreover, postoperative FLS scores did not show any statistically significant difference between the two groups (*p*=0.09). These findings partially agree with a recent study by Liu et al. that demonstrated similar rates of vertigo control but with a trend of poorer FLS scores in MD + M than in MD − M patients following intratympanic gentamicin injection [28]. Therefore, MD + M patients can be treated with similar confidence as their MD − M counterpart when considering ESS for intractable vertigo.

Lastly, our MD + M patients had a shorter duration of major vertigo episodes than MD − M (Table 3). Our study is the first to report this interesting finding. Whether this finding represents a true observation or a Type I false-positive error is unclear given our small and unequal patient population.

Our study had several limitations. It was a retrospective review conducted at a single institution. Over one-third of our initial patient population was excluded from postoperative analysis due to inadequate records. Unfortunately, regular follow-up is often difficult for many of our patients who reside in rural parts of the state and travel a long distance to receive care at our tertiary care center. There was also no control group to account for the natural history of Meniere's disease compared to effect of the intervention.

Another significant limitation was the small patient population. ESS is not a high-volume surgery, so obtaining a large patient population at a single institution is a difficult and common limitation of similar studies. Moreover, the number of migraine patients may be underreported given that the prevalence (18.42%) was similar to the general population, which would be less than expected in patients with Meniere's disease [11, 19].

In our study, migraine is referred to as any form or variant of migraine without a distinction for vestibular migraine. Vestibular migraine is an evolving diagnosis based on a history of migraine and vestibular symptoms, which did not have a consensus definition until 2012 and may occur concurrently with Meniere's disease [2, 15, 17]. If treated today, a subset of our patients may have been also diagnosed with vestibular migraine given that our patients spanned from 1987 to 2019. Hence, migraine treatment was not recorded or mentioned in documentation nor was it recommended by the neurotologist at the time for most of these patients. Ideally, vestibular migraine would have been screened for and treated prior to consideration of ESS, which could be addressed in future studies now that the 2020 Meniere's Disease Guideline recommend screening for it. Nonetheless, we are the first study to account for the migraine comorbidity in Meniere's disease patients undergoing ESS. Further studies are needed to investigate and elaborate on the differences and similarities we observed between the two groups.

## 5. Conclusion

In conclusion, Meniere's disease has a profound impact on patients due to severe vertigo and functional impairment. Migraine is a common comorbidity in those with Meniere's disease. MD + M patients are associated with more psychiatric comorbidities and younger age at the time of ESS than MD − M patients. Nonetheless, ESS appears to have a similarly high efficacy in Meniere's disease with and without migraine.

## Figures and Tables

**Table 1:** All patient demographics stratified by migraine as a comorbidity.

Characteristic	MD + M^a^ (*N* = 14)	MD − M^b^ (*N* = 62)	*P* value [95% CI]
Age (yr)	36.6 ± 15.5	50.8 ± 11.6	0.005^*∗*^^s^ [4.8, 23.6]
Sex			
Male	3 (21.43%)	31 (50.00%)	0.07^∗∗^
Female	11 (78.57%)	31 (50.00%)	
Psychiatric comorbidity	9 (64.29%)	16 (25.80%)	0.01^∗∗^^s^ [1.3, 22.2]
Pure tone average (dB)	49.5 ± 18.6	49.4 ± 15.1	0.99^*∗*^ [−11.4, 11.3]
Word recognition score (%)	69.8 ± 37.2	65.7 ± 32.7	0.70^*∗*^ [−27.0, 18.6]
Preoperative FLS	4.0 ± 1.0	4.3 ± 0.8	0.50^*∗*^ [−0.6, 1.2]
Prior treatment			
Diuretics	11 (78.57%)	49 (79.03%)	0.73^∗∗^
Diuretics + steroids	3 (21.43%)	9 (14.52%)	
Other	0 (0%)	4 (6.45%)	
Meniere's stage		(*N* = 56)^c^	0.35^∗∗^
1: ≤25 dB	3 (21.43%)	4 (7.14%)	
2: 26–40 dB	2 (14.29%)	13 (23.21%)	
3: 41–70 dB	8 (57.14%)	36 (64.29%)	
4: >70 dB	1 (7.14%)	3 (5.36%)	
Diagnosis to surgery (months)	47.43 ± 39.41	52.00 ± 65.61	0.74^*∗*^

^a^Meniere's disease with migraine. ^b^Meniere's disease without migraine. ^c^Population of 56 due to six missing values. ^*∗*^Two-sample *t*-test. ^∗∗^Fisher's exact test. ^S^Significant at 5% level of significance.

**Table 2:** Overall outcomes for endolymphatic sac surgery.

Variable	Preoperative period	Postoperative period	*P* value [95% CI]
Pure tone average (*N* = 42)	50.1 ± 16.2	50.36 ± 21.4	0.91^*∗*^ [−5.2, 4.6]
Word recognition score (*N* = 42)	64.2 ± 34.2	63.2 ± 32.6	0.55^*∗*^ [−5.8, 10.7]
Frequency of major vertigo episodes (per month) (*N* = 48)	16.0 ± 18.5	2.4 ± 5.2	<0.001^*∗*^^s^ [8.1, 19.0]
Functional level scale (*N* = 48)	4.2 ± 0.8	2.8 ± 1.7	<0.001^*∗*^^s^ [0.7, 1.9]
1995 AAO vertigo control (*N* = 48)			
Class A		23 (47.92%)	
Class B		15 (31.25%)	
Class C		1 (2.08%)	
Class D		4 (8.33%)	
Class E		0 (0.00%)	
Class F		5 (10.42%)^∗∗^	

^*∗*^Paired two-sample *t*-test. ^∗∗^Secondary treatment procedures included the following: two patients underwent revision ESS followed by labyrinthectomy, one vestibular nerve section, one revision ESS followed by VNS, and one revision ESS. ^S^Significant at 5% level of significance.

**Table 3:** Outcomes for endolymphatic sac surgery stratified by migraine as a comorbidity.

Variable	MD + M^a^ (*N* = 6^c^)	MD − M^b^ (*N* = 42^c^)	*P* value [95% CI]
Preoperative baseline			
Frequency of major vertigo episodes (per month)	11.2 ± 10.1	16.6 ± 16.7	0.30^*∗*^ [−16.7, 5.6],
Duration of major vertigo episodes (minutes)	143 ± 73.4	393.4 ± 600.7	0.02^*∗*^^s^ [−458.9, −42.0]
Functional level scale	1.8 ± 0.96	2.9 ± 1.83	0.09^*∗*^ [−2.6, 0.25]^*∗*^
1995 AAO vertigo control			
Class A	2 (33.33%)	21 (50.00%)	
Class B	2 (33.33%)	13 (30.95%)	
Class C	0 (0.00%)	1 (2.38%)	
Class D	2 (33.33%)	2 (4.76%)	
Class E	0 (0.00%)	0 (0.00%)	
Class F	0 (0.00%)	5 (11.90%)	
Good control: classes A-B	4 (66.67%)	34 (80.95%)	0.59^∗∗^
Poor control: classes C–F	2 (33.33%)	8 (19.05%)	
Mean vertigo control (%)	32.8 ± 48.9	34.0 ± 65.8	0.96^*∗*^ [−48.4, 51.2]

^a^Meniere's disease with migraine. ^b^Meniere's disease without migraine. ^c^Patients with available data in accordance with time intervals specified by 1995 AAO Guidelines. ^*∗*^Two-sample *t*-test. ^∗∗^Fisher's exact test. ^S^Significant at 5% level of significance.

## Data Availability

Data supporting the results of the study can be obtained upon request to the corresponding author through e-mail.
